# Supplementary material for: Aerobic Exercise Training and In Vivo Akt Activation Counteract Cancer Cachexia by Inducing a Hypertrophic Profile through eIF-2α Modulation
Source: Cancers (Basel). 2021 Dec 22;14(1):28. doi: 10.3390/cancers14010028 (PMC8750332; doi:10.3390/cancers14010028)
Supplement: Supplementary file 1 [file cancers-14-00028-s001.zip › Supplementary Figures.pdf]

Supplemental Figures

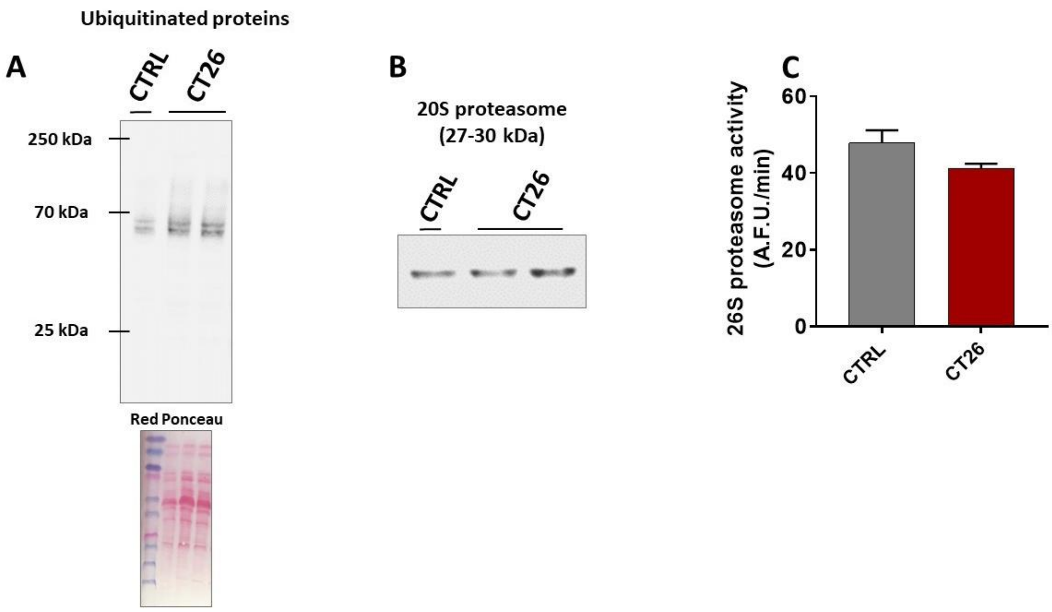

Figure S1. Ubiquitin Proteasome System evaluation in the CT26 model at 17 days post-injection. A: Western Blot of the amount of ubiquitinated proteins. B: Western Blot of 20S proteasome subunit. Red Ponceau was used as loading control. C: 20S proteasome activity. Data are presented as mean ± SD. CTRL, n=4. CT26, n=4.

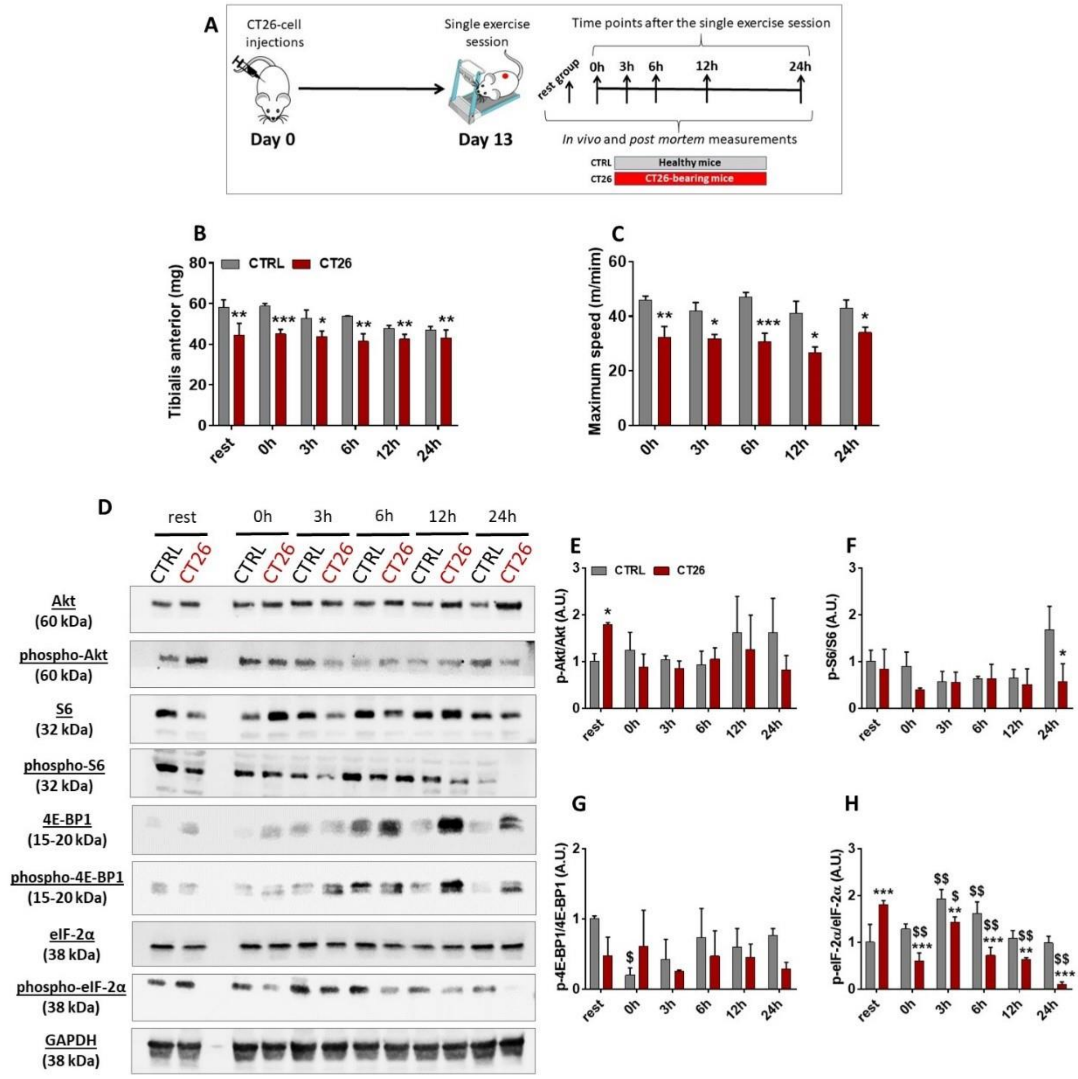

Figure S2. Single exercise session indicates a better translation initiation process as a possible mechanism to control cancer-related muscle wasting. A: Experimental design used to study the effects of a single exercise session in our cancer cachexia model. Mice were previously injected with 10_6 CT26 cells and 13 days later were analysed at different time points. B: TA muscles mass evaluated after 13 days of cell injections, in which can be observed cancer cachexia. C: Maximum speed achieved in the exercise session. Due to the muscle wasting induced by the tumour all the CT26 groups had a worse performance in the session. D: Representative Western Blots images performed on TA muscle. Experimental groups and time points are identified at the top. CTRL: healthy control group; CT26: tumour-bearing mice group. The samples were collected at *rest* and 0h, 3h, 6h, 12h or 24h after the single exercise session. E, F and G: Densitometry analyses of elements of Akt/mTORC1 signalling (Akt, phosphor-Akt$_{ser473}$, S6, phospho-S6$_{ser240/244}$, 4E-BP1 and phospho-4E-BP1$_{thr37/46}$). H: Densitometry analyses of eIF-2α and phospho-eIF-2α_{ser51}. Data are presented as mean ± SD. * p<0.05; ** p<0.01; *** p<0.001 vs. CTRL. $ p<0.05; $$ p<0.01 vs. respective *rest* group. CTRL, n=3-4. CT26, n=3-4.

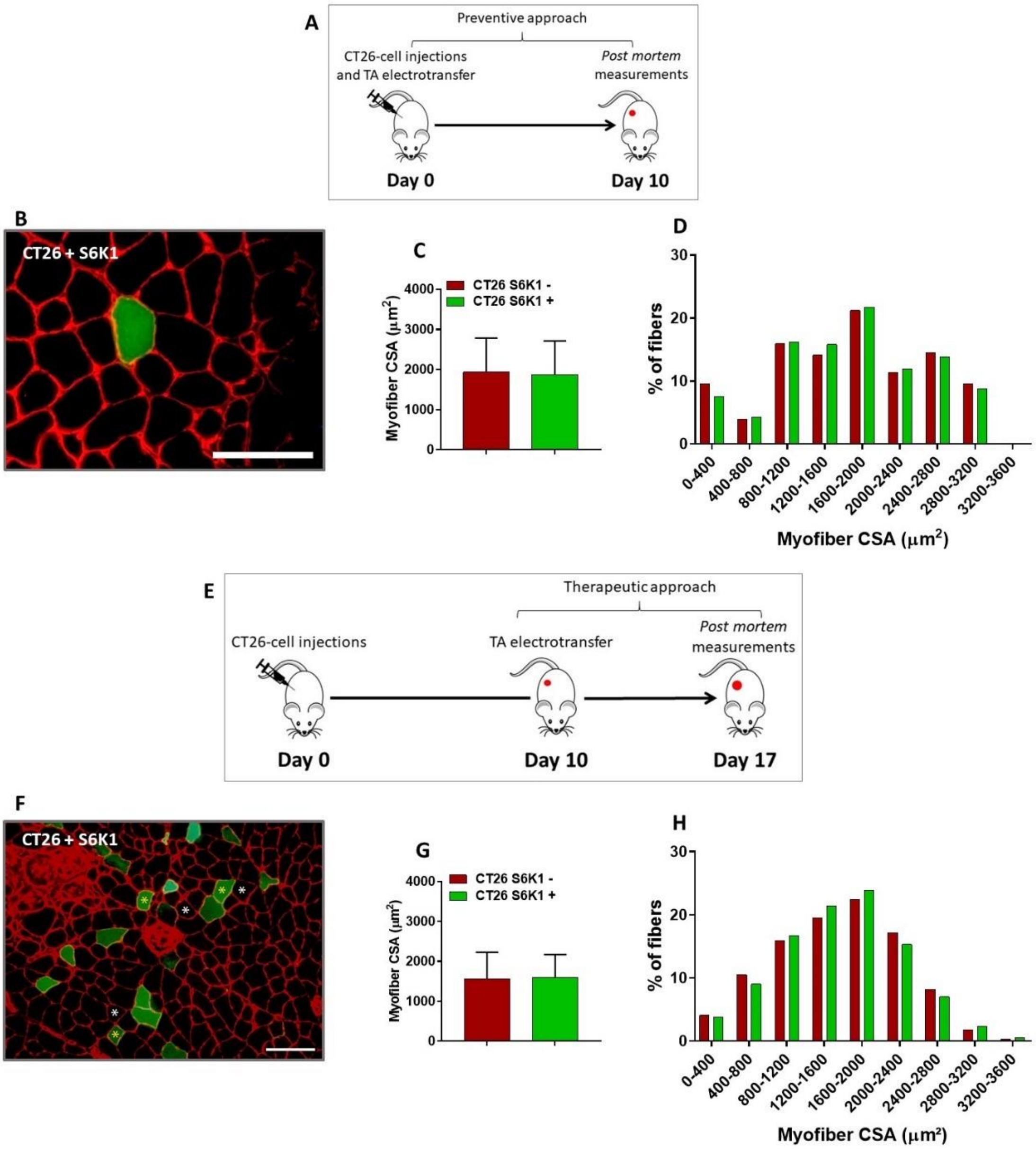

Figure S3. *In vivo* S6K1 activation does not induce hypertrophy in cachectic muscles. A: Experimental design used to determine whether in vivo S6K1 activation could prevent muscle wasting induced by cancer. Mice were injected subcutaneously with 10_6 cells and, at the same day, the TA muscles from right hind limb were electroporated with S6K1 plasmid. Ten days later, muscles were collected and analysed. B: TA muscle cross section immunostained for laminin (red) and GFP (green) showing that the S6K1-positive myofibers (green fibers, indicated by yellow asterisks) are at the same size of that surrounding non-transfected ones (black fibers, indicated by white asterisks). C and D: Myofiber CSA quantification. E: Experimental design used to determine whether S6K1 eletroporation could revert cachexia. Mice were injected subcutaneously with 10_6 cells and 10 days later the TA muscles from right hind limb were electroporated with S6K1. Seven days later, muscles were collected and analysed. F: TA muscle cross section immunostained for laminin (red) and GFP (green). The S6k1-positive myofibers (green fibers, indicated by yellow asterisks) are at the same size of the surrounding non-transfected ones (black fibers, indicated by white asterisks). G and H: Myofiber CSA quantification. *CT26 S6K1 +*: S6K1-positive TA myofibers from CT26-bearing mice; *CT26 S6K1 -*: S6K1-negative TA myofibers from CT26-bearing mice. Scale bar: 100 μm. Data are presented as mean ± SD. CT26, n=4.

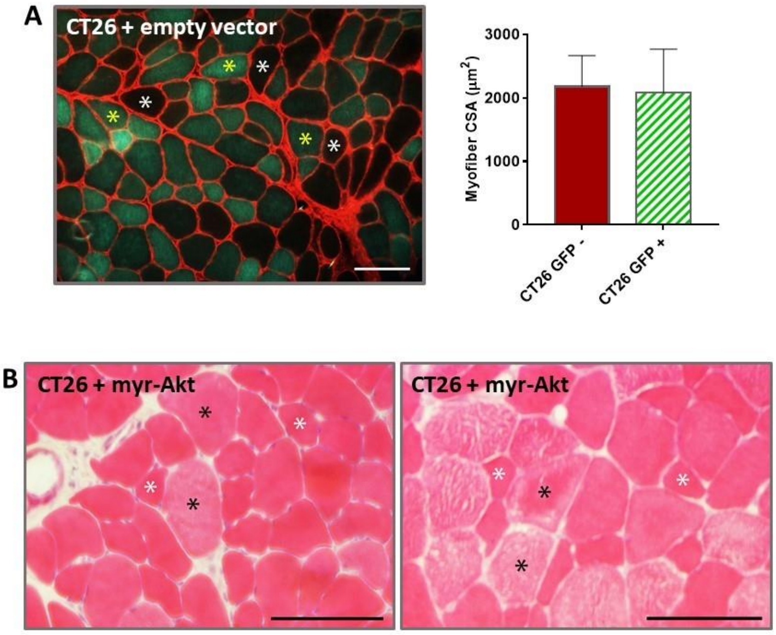

Figure S4. A: Left TA muscle cross section from CT26-bearing mouse 10 days after tumour cells injection and plasmids electroporation immunostained for laminin (red) and GFP (green) and CSA quantification. Note that the Snap-GFP-positive myofibers (indicated by yellow asterisks) presented the same size of the surrounding non-transfected ones (indicated by white asterisks). It shows that Snap-GFP plasmid does not affect the myofiber CSA. Scale bar: 100 μm. B: TA muscle cross section from CT26-bearing mouse in the end of the therapeutic intervention, i.e., 7 days after Akt electroporation and 17 days after tumour cells injection. The cross sections were H&E stained. Note that the Akt-positive myofibers (indicated by black asterisks) are bigger than the surrounding non-transfected ones (indicated by white asterisks). Scale bar: 100 μm. Data are presented as mean ± SD.
