# Supplementary material for: Aerobic Exercise Training and In Vivo Akt Activation Counteract Cancer Cachexia by Inducing a Hypertrophic Profile through eIF-2α Modulation
Source: Cancers (Basel). 2021 Dec 22;14(1):28. doi: 10.3390/cancers14010028 (PMC8750332; doi:10.3390/cancers14010028)
Supplement: Supplementary file 1 [file cancers-14-00028-s001.zip › Supplementary File S1.pdf]

1 – Uncropped western blot of Figure 2 – Aerobic exercise training delays cancer cachexia progression.

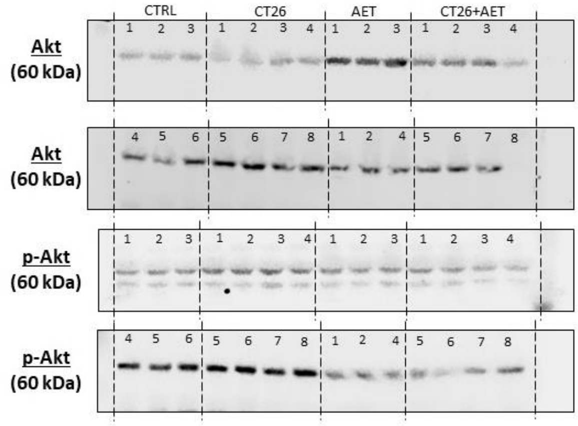

Akt/p-Akt	1	2	3	4	5	6	7	8
CTRL	1,44	1,64	1,57	1,22	1,62	1,23		
CT26	2,63	2,10	2,00	1,63	1,96	2,43	2,10	2,20
AET	1,08	1,13	0,96	0,90				
CT26+AET	1,37	1,04	0,75	0,84	1,01	0,96	0,83	1,01

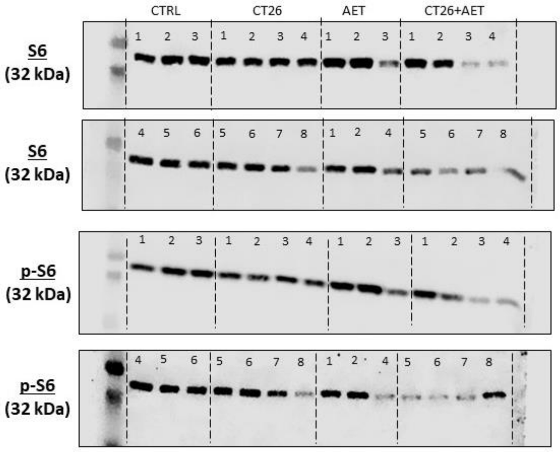

S6/p-S6	1	2	3	4	5	6	7	8
CTRL	1,06	0,95	0,98	1,12	0,98	1,03		
CT26	1,05	1,17	1,12	1,17	1,09	1,00	0,78	1,02
AET	1,01	0,97	0,86	0,90				
CT26+AET	0,99	0,79	0,95	0,68	0,66	0,55	0,80	0,86

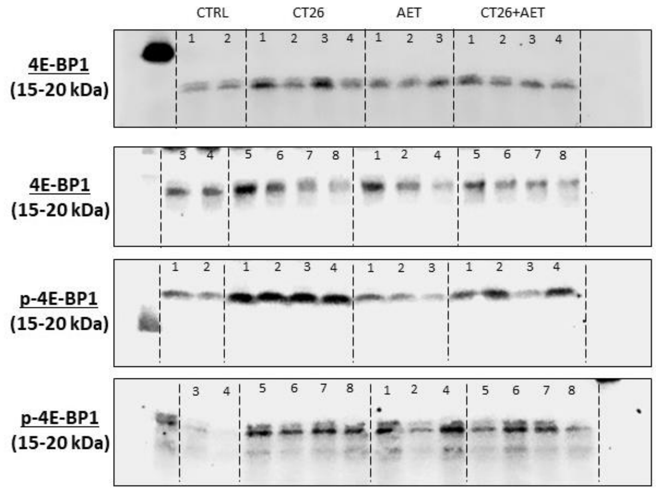

4E-BP1/p-4E-BP1	1	2	3	4	5	6	7	8
CTRL	1,26	0,68	0,87	0,53	1,44	1,37		
CT26	1,08	2,21	1,19	2,24	1,08	1,95	2,88	1,76
AET	0,88	0,56	0,20	0,58				
CT26+AET	0,62	1,46	0,40	1,45	0,77	2,03	0,58	2,48

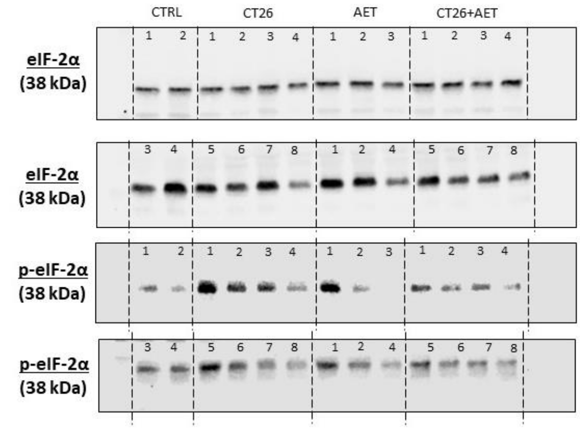

eIF-2a/p-eIF-2a	1	2	3	4	5	6	7	8
CTRL	0,49	0,30	0,29	0,51	0,37	0,29		
CT26	1,14	0,90	0,70	0,60	0,88	0,74	0,92	0,70
AET	0,82	0,28	0,39	0,27				
CT26+AET	0,52	0,43	0,46	0,28	0,39	0,41		

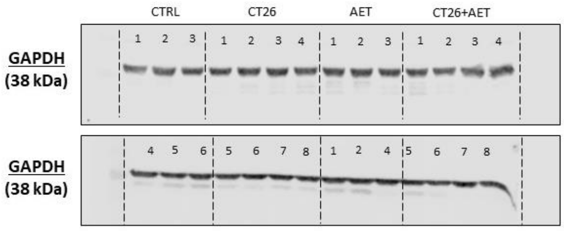

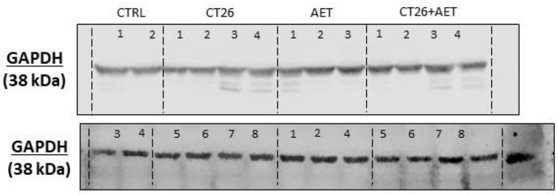

2 – Uncropped western blot of Figure S2 – Single exercise session indicates a better translation initiation process as a possible mechanism to control cancer-related muscle wasting.

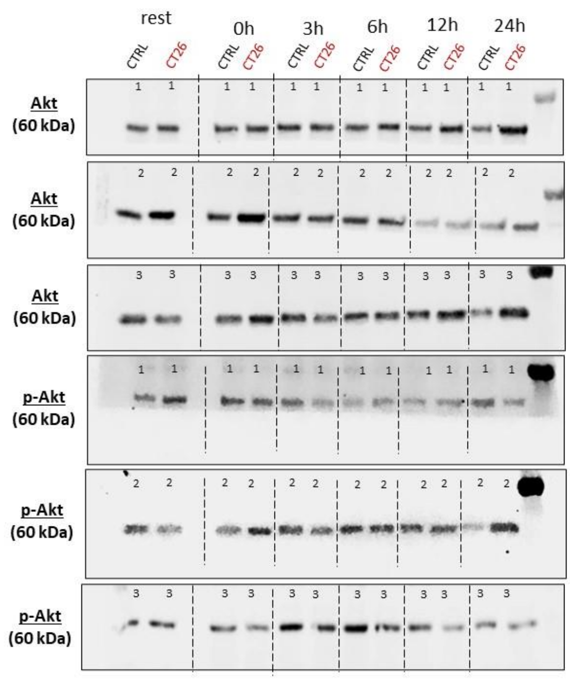

Akt/p-Akt	rest		0h		3h		6h		12h		24h	
	CTRL	CT26	CTRL	CT26	CTRL	CT26	CTRL	CT26	CTRL	CT26	CTRL	CT26
1	1,23	1,82	1,56	1,18	1,15	0,79	0,85	0,95	1,11	0,85	1,95	0,48
2	0,90	1,82	1,42	0,60	0,99	0,75	0,68	0,91	2,57	2,16	2,21	1,05
3	0,93	0,77	0,82	0,93	1,06	1,07	1,29	1,36	1,29	0,88	0,80	1,00

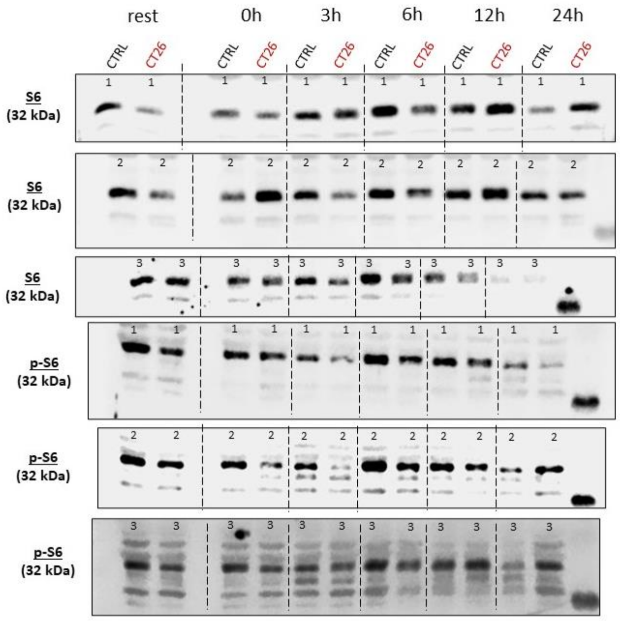

S6/p-S6	rest		0h		3h		6h		12h		24h	
	CTRL	CT26	CTRL	CT26	CTRL	CT26	CTRL	CT26	CTRL	CT26	CTRL	CT26
1	1,48	2,22	1,76	0,70	0,70	0,92	1,11	1,17	0,98	0,62	2,22	1,42
2	2,08	1,20	1,18	0,70	0,72	0,92	0,93	0,40	0,73	0,23	3,43	0,52
3	1,19	0,78	1,03	0,58	0,91	0,46	1,13	1,05	1,24	1,50	2,83	0,97

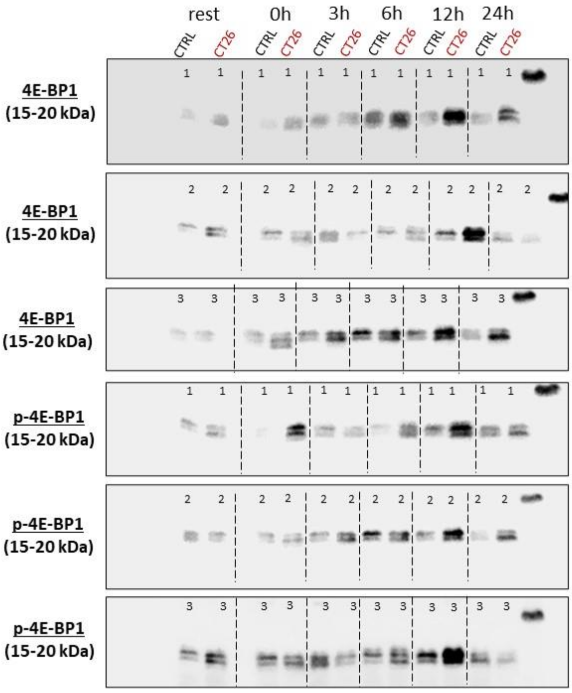

4E-BP1/p-4E-BP1	rest		0h		3h		6h		12h		24h	
	CTRL	CT26	CTRL	CT26	CTRL	CT26	CTRL	CT26	CTRL	CT26	CTRL	CT26
1	2,60	1,25	0,75	3,52	1,00	0,76	0,85	0,57	1,80	0,94	2,12	0,70
2	2,78	2,15	0,25	0,72	0,58	0,62	2,00	1,09	2,25	2,02	1,80	1,14
3	2,86	1,45	0,72	2,05	2,35	0,70	3,17	2,76	2,03	1,17	2,38	0,62

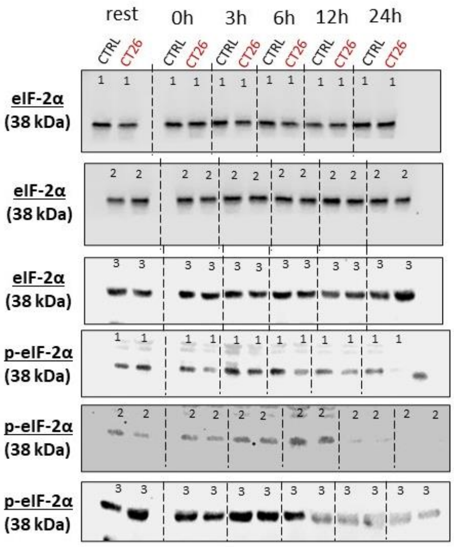

eiF-2a/p-eiF-2a	rest		0h		3h		6h		12h		24h	
	CTRL	CT26	CTRL	CT26	CTRL	CT26	CTRL	CT26	CTRL	CT26	CTRL	CT26
1	0,30	0,75	0,50	0,20	0,80	0,63	0,60	0,25	0,50	0,25	0,36	0,03
2	0,53	0,74	0,57	0,30	0,80	0,56	0,74	0,35	0,40	0,28	0,46	0,06
3	0,41	0,75	0,53	0,25	0,80	0,59	0,67	0,30	0,45	0,26	0,41	0,04

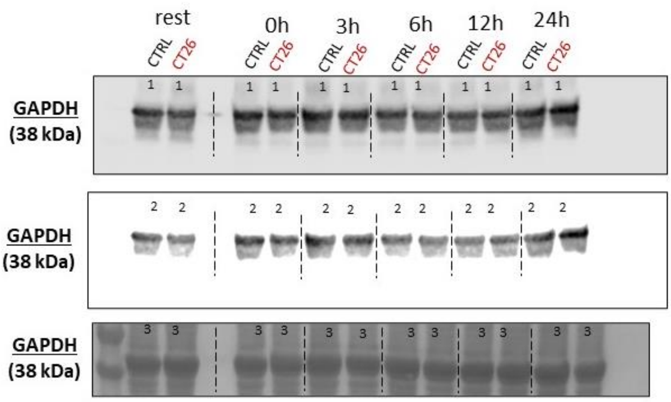

3 – Uncropped western blot of Figure 4 – Functional and hypertrophic adaptations induced by the Akt activation in cachectic muscles.

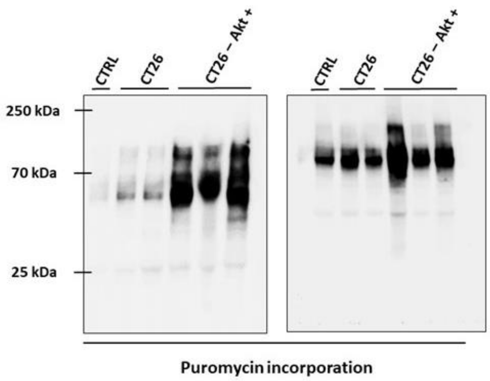

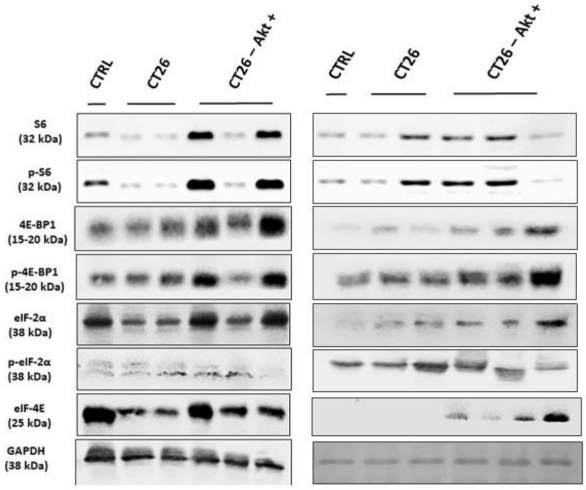

	CT26	CT26	CT26 Akt+	CT26 Akt+	CT26 Akt +
S6/p-S6	0,48	0,39	1,02	0,42	0,99
4E-BP1/p-4E-BP1	1,20	1,20	1,49	0,70	1,03
eiF-2a/p-eiF-2a	1,46	1,83	0,83	1,48	0,39
eiF4E	0,52	0,47	0,96	0,58	0,64
